# Significance of alpha smooth muscle actin expression in traumatic painful neuromas: a pilot study in rats

**DOI:** 10.1038/srep23828

**Published:** 2016-03-29

**Authors:** Weidong Weng, Bin Zhao, Dingshen Lin, Weiyang Gao, Zhijie Li, Hede Yan

**Affiliations:** 1 Department of Orthopaedics (Division of Plastic and Hand Surgery) The Second Affiliated Hospital of Wenzhou Medical University, Wenzhou, China

## Abstract

Treatment of painful neuromas remains a challenge and the mechanism of neuroma-associated pain is not yet fully understood. In this study, we aimed to observe the expression of alpha smooth muscle actin (α-SMA) in traumatic neuromas and to investigate its possible roles in the cause of neuropathic pain in a rat model. The rat sciatic nerve was used and the experiment was divided into two parts. In part I, our results showed significantly higher levels of α-SMA and the pain marker c-fos in the autotomy group than in the no-autotomy group. In part II, the expression of α-SMA in neuromas was down- and up-regulated using SB-431542 and GW9662, respectively. A significant correlation between autotomy scores and the expression level of α-SMA was found (R = 0.957; p < 0.001) and the expression level of α-SMA was positively related to the autotomy scores (R^2^ = 0.915, p < 0.001). We concluded that the expression of α-SMA plays certain roles in the neuroma-associated pain, either as a direct cause of pain or as an indirect marker of existence of local mechanical stimuli. Our findings may provide new insights into the development of new treatment modalities for the management of intractable painful neuromas.

The transection of a peripheral nerve, especially in cases of traumatic amputation of a limb, is followed almost invariably by the formation of a traumatic neuroma which leads to incapacitating pain in about 10% of patients^1^. Several theories have been proposed to explain the mechanism or pathophysiology of this neuropathic pain, including the gate theory, the persistent mechanical or chemical irritation of the axons, (and/or) persistent stimulation of the axons within the neuroma via spontaneous discharge^2^. However, the exact mechanism of neuroma-associated pain is not yet fully understood, contributing to the challenge of managing patients with painful neuromas^3^,^4^.

Pathologically, the traumatic neuroma is usually in a bulbous shape, consisting of an unorganized network of connective tissue intermingled with nerve fibers, Schwann cells, macrophages, fibroblasts, and myofibroblasts, the latter thought to contribute to pain causing the collagen matrix to contract around nerve fibers^5^. In our previous study^6^, obviously positive staining of alpha smooth muscle actin (α-SMA) was found in the traumatic neuromas collected from the patients suffering from neuropathic pain. Furthermore, the expression intensity of α-SMA was positively related to the scores of visual analogue scale (VAS). We, therefore, postulated that its expression may contribute to neuroma-associated pain either as a direct cause of pain or an indirect marker of the existence of local mechanical stimuli. In this experimental study, we aim to further investigate the significance of α-SMA expression in the traumatic neuroma and potential role in the mechanism of neuropathic pain in a rat model.

## Results

### Results of Part I

#### Animal selected

Of the sixty rats, one died 10 days after surgery; seventeen (no-autotomy group) showed no evidence of autotomy and the other 42 animals (autotomy group) all developed varying degrees of autotomy at 4 weeks after surgery. Twelve animals were randomly selected using random number table method from each group for the following studies. The rats left (n = 35) that were not selected for the following studies in both groups were provided for microsurgical training for our postgraduate students.

#### Gross evaluation

The neuroma in the no-autotomy group had a slightly lower WR (0.63 ± 0.051) in comparison with that of the autotomy group (0.66 ± 0.065); however, no significant difference of WR was seen between the two groups (p = 0.242). In contrast, adhesion scores varied between them. Compared to the no-autotomy group (1.58 ± 0.67), the autotomy group (2.25 ± 0.62) showed significantly higher scores (p = 0.022).

#### Expression level of α-SMA in neuromas

**Figure 1:**
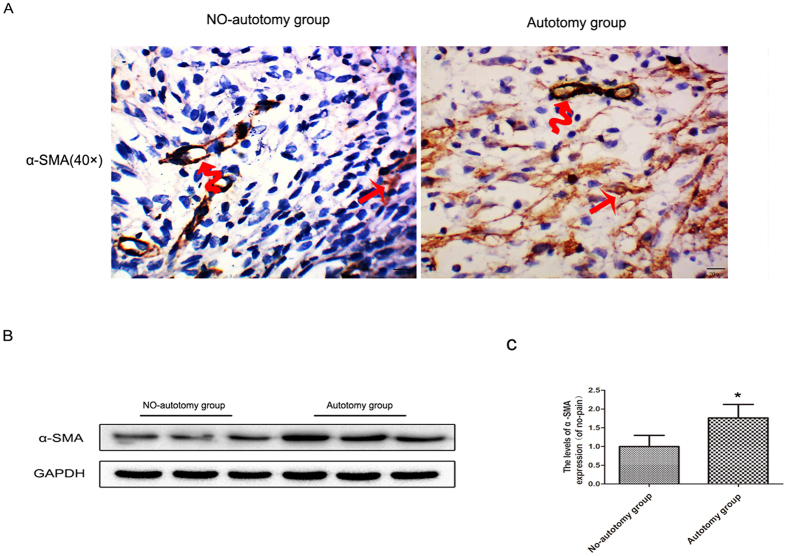
Increased level of α-SMA in the neuromas in the autotomy group. (**A**) Immunohistochemistry analysis of α-SMA expression in the two groups at 4 weeks after transection (original magnification × 400). In the no-autotomy group, only slightly positive expression of α-SMA was seen; while in the autotomy group, extensively positive expression of α-SMA was present. The curved arrow shows the highly stained blood vessels and the straight arrow shows one of the positively stained myofibroblasts. (**B**) Protein expressions of α-SMA in the neuromas. GAPDH was used as the loading control and for band density normalization. (**C**) The optical density analysis of α-SMA proteins. Values are expressed as the mean ± SEM, n = 6 per group. *P < 0.05 versus the no-autotomy group.

The immunostaining results showed that significant positive staining of α-SMA was noted in the autotomy group. The area of staining was located in the cytoplasm of the proliferative myofibroblasts and distributed diffusely in the regenerated nerve fibers. In contrast, only slightly positive staining of α-SMA was noted in the no-autotomy group (Fig. 1A). These findings were in line with the results of Western-blot assay, which showed a significantly higher level of α-SMA in the autotomy group in comparison with that of the no-autotomy group (*vs. autotomy group p < 0.05; Fig. 1B,C).

#### Expression level of c-fos in the dorsal horn of L4 spinal cord

**Figure 2:**
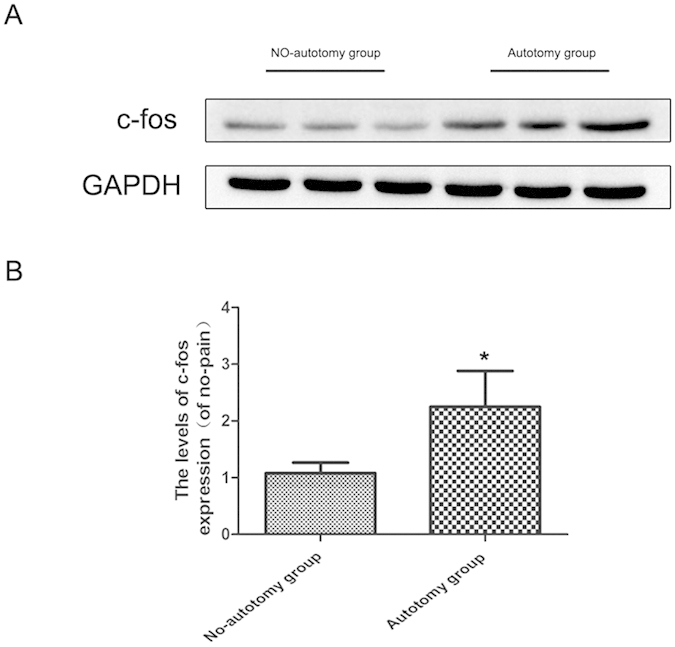
Relative expression of c-fos in the dorsal horn of L4 spinal cord. (**A**) Protein expression of c-fos of rats in no-autotomy group, autotomy group respectively. GAPDH was used as the loading control and for band density normalization. (**B**) The optical density analysis of c-fos in two groups. Values are expressed as the mean ± SEM, n = 6 per group. *P < 0.05 versus the no-autotomy group.

The expression of c-fos in the dorsal horn of the fourth lumbar spinal cord was significantly higher in the autotomy group than in the no-autotomy group (*vs. autotomy group p < 0.05; Fig. 2).

### Results of Part II

#### Behavior assessment

**Figure 3:**
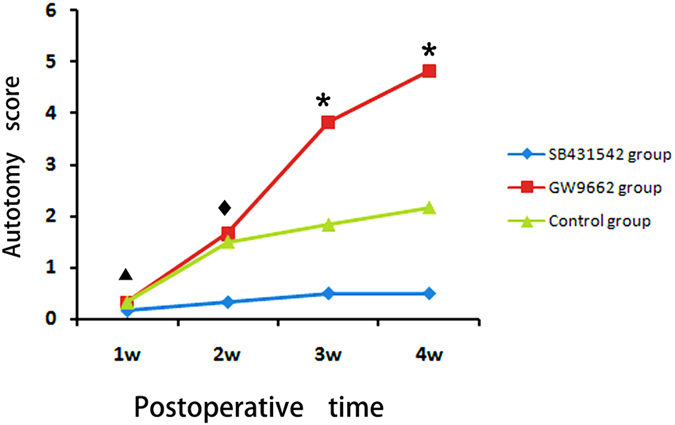
Results of weekly average autotomy scores. Significant differences in the average autotomy score were observed among the three groups two weeks after surgery (*p < 0.05) except at the end of the first week (^▲^p > 0.05: SB-431542 vs. control group, p = 0.818; GW9662 vs. control group, p = 0.645; SB-431542 vs. GW9662, p = 0.490) and between GW9662 group and control group at 2 weeks (^♦^p = 0.25).

Autotomy behavior was all noted on the operated side. Significant differences in the average autotomy score were observed among the three groups two weeks after surgery (*p < 0.05; Fig. 3). In addition, the occurrence of autotomy in the SB-431542 group was the lowest and in the GW9662 group the highest of the three groups: all the animals in the GW9662 group (6/6, 100%) developed varying degrees of autotomy (autotomy score 3–7, 5.33 ± 1.37); in contrast, only 1 rat in the SB-431542 group (1/6, 16.7%, autonomy score: 1) and 3 rats in the control group (3/6, 50%, autotomy score: 2–6, 2.17 ± 1.11) showed evidence of autotomy.

#### Gross evaluation

The neuroma in the SB-431542 group had a lower WR (0.48 ± 0.079) than in the control group (0.61 ± 0.049) while the highest WR was seen in the GW9662 group (0.72 ± 0.041); the differences in WR were significant among the three groups (SB-431542 vs. control group, p = 0.002; GW9662 group vs. control group, p = 0.008; SB-431542 vs. GW9662, p < 0.001). The average adhesion scores of the SB-431542, GW9662 and control group were 1.17 ± 0.41, 2.67 ± 0.52 and 1.83 ± 0.41, respectively. Furthermore, significant differences in adhesion scores were observed among the three groups (SB-431542 vs. control group, p = 0.021; GW9662 vs. control group, p = 0.006; SB-431542 vs. GW9662, p < 0.001).

#### Expression level of α-SMA and correlation with the autotomy scores

**Figure 4:**
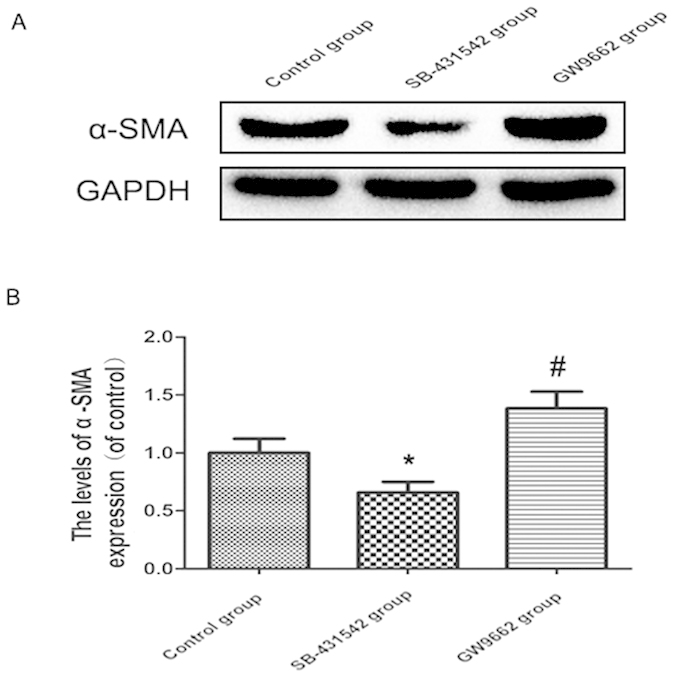
Effects of SB-431542 and GW9662 on the expression of α-SMA in the neuromas. (**A**) Protein expression of α-SMA in control, SB-431542 and GW9662 groups, respectively. GAPDH was used as the loading control and for band density normalization. (**B**) The optical density analysis of α-SMA protein in three groups. Values are expressed as the mean ± SEM, n = 6 per group *P < 0.05 versus the control group, ^#^P < 0.05 versus the control group.

As expected, the results of Western-blot assay showed that the expression of α-SMA was significantly up-regulated in the GW9662 group and down-regulated in the SB-431542 group in comparison with that of the control group (all p < 0.05; Fig. 4).

**Figure 5:**
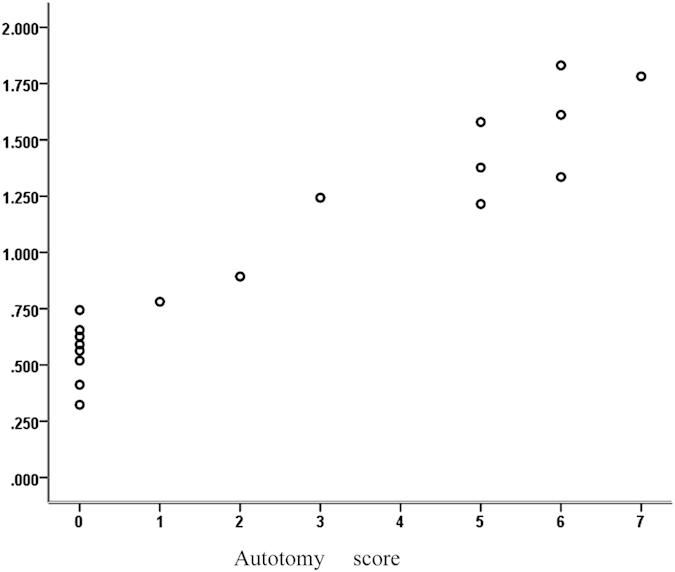
Correlation between autotomy scores and the expression levels of α-SMA. The scatter diagram demonstrated a positive correlation between the autotomy scores and expression levels of α-SMA (R^2^ = 0.915, p < 0.001).

Pearson correlation analysis demonstrated a significant correlation between autotomy scores and the expression level of α-SMA (R = 0.957; p < 0.001). Linear regression indicated that the expression level of α-SMA was positively related to autotomy scores (R^2^ = 0.915, p < 0.001; Fig. 5).

#### Expression of c-fos in the dorsal horn of L4 spinal cord

**Figure 6:**
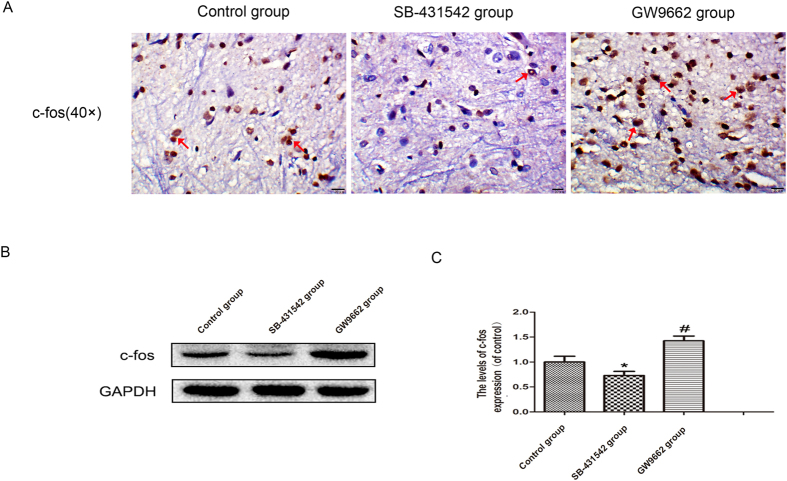
Effects of SB-431542 and GW9662 on the expression of c-fos in the dorsal horn of L4 spinal cord. (**A**) c-fos expression in each group as assessed by immunohistochemistry (original magnification × 400). Significantly positive staining of c-fos in the neuron cells was seen in GW9662 group; in contrast, slightly positive staining of c-fos in the neuron cells was observed in the control group; while only very few neuron cells had positive staining of c-fos in the SB-431542 group. The arrows showed the neuron cells with positive staining of c-fos. (**B**) Protein expression of c-fos in control, SB-431542 and GW9662 groups, respectively. GAPDH was used as the loading control and for band density normalization. (**C**) The optical density analysis of c-fos protein in three groups. Values are expressed as the mean ± SEM, n = 6 per group *P < 0.05 versus the control group, ^#^P < 0.05 versus the control group.

The results of immunohistological analysis of c-fos showed significant positive staining of c-fos in the neuron cells of group GW9662. In contrast, slightly positive staining of c-fos in the neuron cells was observed in the control group; while only very few neuron cells had positive c-fos staining in group SB-431542 (Fig. 6A). The expression level of c-fos in the dorsal horn of the fourth lumbar spinal cord was lowest in the SB-431542 group, and highest in the GW9662 group (all p < 0.05; Fig. 6B,C).

## Discussion

In clinical settings, patients are able to report pain levels. However, researchers have found it difficult to evaluate the pain status in experimental animals^7^,^8^. As an alternative, the behavior of autotomy, which is triggered by neurotomy of peripheral nerve in animals and charactered as the presentation of a typical behavior of licking, scratching and self-mutilation of the denervated limb, has been regarded as an animal model of neuropathic pain-related spontaneous sensory disorders^9^,^10^. However, no studies so far have convincingly confirmed whether or not the observed behavior was directly related to pain^8^. Quantitative assessment of tactile allodynia^11^ or thermal hyperalgesia^12^ in rat paws has been used as a simple method of evaluating neuropathic pain in research models; however, the occurence of autotomy in the studied limb precludes these methods. In the literature, c-fos was reported as a pain-related protein^8^,^13^. In this study, our findings showed that the expression level of c-fos was significantly higher in the autotomy group in comparison with the no-autotomy group, indicating that the status of autotomy behavior may be a potential indicator of pain intensity.

Myofibroblasts are the matrix-producing interstitial reactive cells that occur after acute or chronic injury^14^ and α-SMA is a marker of the myofibroblastic phenotype^15^. Myofibroblasts are highly contractile^16^,^17^ and have been widely considered to actively promote dermal wound contraction^18^. In addition, α-SMA contributes to the additional generation of contractile forces in non-muscle cells and can upregulate the contractile activity of fibroblasts^19^,^20^. The contractile activity of myofiroblasts is beneficial for dermal wound closure and for restoring the mechanical stability of injured organs against rupture^9^.

However, the above mentioned biological characteristics of myofibroblasts (α-SMA) may “entrap” regenerated nerve fibers and cause spontaneous pain. In our previous study^6^, we observed that there was positive staining of α-SMA in the traumatic neuromas harvested from the patients with neuropathic pain and the expression intensity of α-SMA was positively correlated to the scores on the visual analogue scale. In this experiment, a significant positive expression of α-SMA was also present in the autotomy group. In contrast, only slightly positive staining was detected in the no-autotomy group. Our further study demonstrated that the autotomy score and c-fos expression were both significantly decreased by down-regulation of α-SMA expression with SB-431542. On the other hand, up-regulation of α-SMA expression with GW9662 contributed to higher autotomy scores and increased c-fos expression. Importantly, a significant correlation between the autotomy score and the expression level of α-SMA was observed. Therefore, we speculated that the contractile myofibroblast may entrap the regenerated small nerve fibers within the neuroma, leading to persistent mechanical stimulation of the regenerated nerve fibers, resulting in the occurrence of autotomy in the autotomy group. Presumably, based on the inherent self-contractile characteristic of α-SMA, this type of pain might be considered as a spontaneous one.

On the other hand, investigations have shown that exogenously applied and endogenously generated mechanical tension can regulate α-SMA expression, and its expression positively responds to the mechanical tension^21^. α-SMA is also considered as not only a mediator of traction forces in contractile myofibroblasts, but also as a component of the global mechanotransduction system^22^. Coincidently, significantly higher adhesion scores were observed in the autotomy group compared to the no-autotomy group in the first part of this study. Furthermore, much higher adhesion scores were seen in the GW9662 group in comparison to the SB-431542 and control groups in the part II study. These findings suggested the presence of more local mechanical stimuli around neuromas in the autotomy group and GW9662 groups.

In clinical practice, our findings may provide certain rationale for the widely accepted approach in the treatment of painful neuroma by transferring the nerve ends into muscles or bones or capping the nerve ends with veins or nerve conduits^23^,^24^,^25^. It is conceivable that the external mechanical stimuli can be reduced by capping the nerve ends with these methods and away from the scar tissues, and accordingly the expression of α-SMA in the neuroma may be decreased, contributing to the relief of pain and discomfort^6^. From this perspective, the persistent expression of α-SMA in the autotomy group may be a result of the persistent local mechanical stresses caused by the adhesive scar tissue surrounding neuromas. We might take this type of pain as an evoked pain due to local external mechanical stimulus.

Therefore, our findings strongly suggest that α-SMA plays a role in the pathology of traumatic neuromas, either as a direct cause of neuroma-associated spontaneous pain (internal compression), or an indirect marker of the existence of local mechanical stimuli (external compression). However, a solid conclusion regarding the exact role of α-SMA in the neuropathic pain still can’t be drawn based on our present findings and further research is warranted.

Studies have demonstrated that inhibition of neuroma growth benefited the treatment and prevention of neuropathic pain in the management of traumatic painful neuromas^26^,^27^,^28^,^29^. In part II of this study, a lower WR in the SB-431542 group seemingly contributed to deceased severity of pain and a higher WR in the GW9662 group resulted in a higher pain status. However, in the part I study no significant difference in neuroma growth was seen between the autotomy group and no-autotomy group. These inconsistent results seem to suggest that the neuropathic pain may not be closely associated with the volume of the neuroma mass. Further studies are required to gain insight into these seemingly contradictory findings.

As a pilot study, one concern of this research is why, in the first phase of this study, some animals from the same series developed pain while others did not. This may be caused by differences in technique between the two surgeons, possibly leading to different amounts of adhesion at the operative site. However, in clinical settings it is not unusual to see a wide variation in pain response between individuals who have received similar nerve injuries. Therefore, we should not be surprised to see similar variations within our rat model population. This fact highlights the complexity of traumatic neuropathic pain. Follow-up studies are warranted to further confirm the role of α-SMA and gain insights into its pain mechanism in traumatic neuromas.

In summary, the expression of α-SMA seems to play a role in neuroma-associated pain, possibly either as a direct cause of pain or as an indirect marker of the existence of local mechanical stimuli. The finding of significantly higher adhesion scores and positive expression of α-SMA in the autotomy group provide support to the basic rationale for the treatment modalities of capping or burying a painful neuroma in surrounding tissues to prevent scar tissue adhesion and reduce local mechanical stimulus in the management of painful neuromas. Furthermore, the positive correlation between the expression level of α-SMA and severity of pain in traumatic neuromas may contribute to the future development of a practical treatment strategy by inhibiting the expression of α-SMA. Clearly, more studies focusing on the exact role of α-SMA in the pathology of neuropathic pain are needed to provide evidence-based information.

## Materials and Methods

### Animal use

This investigation was performed under a protocol approved by the Institutional Animal Care Committee, Wenzhou Medical University. All experiments used in this study received humane care in accordance with the guidelines of the National Research Council for the care and use of laboratory animals. Seventy-eight male Sprague-Dawley rats weighing 250 to 300 g were purchased from Wenzhou Medical University Centre for Laboratory Animals. The animals were kept in specific, pathogen-free conditions in 12-hr day–night cycles at 22–24 °C. All rats received food and water *ad libitum.*

### Surgical procedure and animal model preparation

The Animals were anaesthetized with an intraperitoneal injection of pentobarbital sodium (50 mg/kg), and placed in the prone position. An incision was made in the right thigh, and the right sciatic nerve was exposed between the biceps femoris and the gluteal muscles. Under the surgical microscope, the target site giving off the branch of the posterior gluteal nerve (PGN) was marked with a 7–0 suture to conduct a quantitative analysis for neuroma growth. Then, the sciatic nerve was sharply transected 1 cm distal to the marked site with the assistance of a 10 mm -long length marker and a 15 mm-long piece of the distal segment of the transected nerve was removed to prevent nerve regeneration. Muscles and skin were closed with 4–0 sutures in layers and the animals were placed back in separate cages. Ibuprofen analgesia was administered daily for 1 week postoperatively in all groups. All surgical procedures were carried out by the same team of two surgeons.

The experiment was carried out in two phases with the following specific objectives: Part I: Observe the expression status of α-SMA in traumatic neuromas in rats with and without autotomy; Part II: Evaluate the impact of intervention on the expression level of a-SMA and the severity of pain.

## PART I

At 4 weeks after transection, the autotomy status of rats (n = 60) was evaluated by observation of autotomy behavior. Based on the presence or absence of autotomy, the animals were divided into two groups: no-autotomy group and autotomy group. According to the experimental protocol, twelve rats in each group were randomly selected and used for the following analysis.

### Specimen preparation

The rats in each group were sacrificed by administering an overdose of sodium pentobarbital solution. The proximal nerve stump was cut from the level of the marked site (the origin of PGN) along with a 1 cm-long segment of normal nerve from the corresponding part on the left side. Half of the specimens in each group were randomly selected for immunohistological study (n = 6) and the other half were used for the detection of α-SMA levels. In addition, the dorsal horn of the fourth lumbar spinal cord (positioned by the L 4 nerve root) was collected for western blot analysis of the expression of the pain marker c-fos^8^,^13^. All samples were stored at −80 °C until analyses were performed.

### Gross evaluation

Neuroma adhesion was scored by an investigator who was blind to the pain status of the animal. Perineural adhesions around the neuroma were classified into four categories: score 0: the neuroma is mobile and free of adhesion; 1, mild: the neuroma can be isolated easily with forceps; 2, moderate: blunt dissection with hemostats is required for mobilization; 3, severe: the neuroma is unable to be isolated without sharp dissection. Weight ratios (WR) were used as an index of neuroma growth and were calculated using the following equation: [the weight of neuroma (NW) – the weight of the excised normal nerve segment (NNW)]/NNW × 100.

### Histologic analysis

The excised proximal stumps were first weighed and then cut at a level 5 mm distal to the proximal normal end, leaving the neuroma for histological study. The nerve specimens were then fixed in 10% formalin, embedded in paraffin wax and cut into 4-μm sections. In order to standardize the site for histological staining, the sections were randomly selected at a distance ranging from 400 to 600 um from the distal ending of the specimens. At least 30 sections were obtained from each sample after small sections or poorly cut sections were excluded, leaving at least 20 sections from which 5 were randomly selected. Monoclonal antibodies against the following proteins were used: the monoclonal rabbit anti-mouse a-SMA (1:200, Sigma, USA), monoclonal rabbit anti-mouse c-fos (1:200, Sigma, USA), and secondary monoclonal goat anti-rabbit antibody (1:500, Sigma, USA). Streptavidin–peroxidase (SP) immunohistochemical staining was performed. To examine the specificity of immunostaining, phosphate-buffered saline (PBS) was used to replace the primary antibody as the control and the specificity was further verified by the immunohistochemical response of the small vessels in all the sections for the staining of a-SMA. The slides were observed under a light microscope, and brown-yellow color staining was interpreted as positive expression.

### Western Blot Analysis

For extraction of total cellular protein, the tissue was homogenized in a modified lysis buffer (100 mmol/L dithiothreitol, 50 mmol/L Tris-HCl, pH 6.8, 2% SDS, and 10% glycerol) containing protease inhibitors. Protein concentration was measured using a bicinchoninic acid protein assay, and total protein was kept at −80 °C until use. Equal protein was separated by SDS-PAGE and then transferred onto a PVDF membrane.

After being incubated with 5% non-fat milk in TBS with 0.05% Tween 20 for 1 h, blots were incubated with monoclonal antibodies against rabbit anti-α-SMA (1:1000,Abcam, Cambridge, MA) and rabbit anti-c-fos (1:000,Abcam, Cambridge, MA) overnight at 4 °C. The membranes were washed with TBS three times and incubated with HRP-conjugated goat anti-rabbit IgG (1:8000) for 2 h at room temperature. Signals were visualized using the Chemi- DicTM XRS + Imaging System (Bio-Rad). The level of analyzed protein was normalized to that of GAPDH (1:800, Santa Cruz Biotechnology, USA).

## PART II

In order to further investigate the correlation between the expression level of a-SMA in the traumatic neuroma and the severity of pain, the intervention using SB-431542 and GW9662 was carried out. The SB-431542 is a TGFβ1 receptor inhibitor, which has been widely utilized and found to be reliable in down-regulation of α-SMA expression *in vivo* and *in vitro*^30^,^31^. While the GW9662 is a generally accepted PPARγ antagonist, which can up-regulate the expression of α-SMA and aggravate the process related to fibrogenesis *in vivo* and *vitro*^32^,^33^.

### Drugs and chemicals

SB-431542 (Selleck Chemicals, Houston, TX, USA) and GW9662 (Selleck Chemicals, Houston, TX, USA) was dissolved in DMSO and further diluted in saline for intraperitoneal injection.

### Experimental protocol

The same surgical procedure as part I was utilized. The rats (n = 18) were randomly assigned into three groups according to the intervention: (1) SB-431542 group (n = 6): After surgery, the animals received SB-431542 (3 mg/kg, i.p, single dose) treatment on the 1^st^, 3^rd^, 5^th^, 7^th^, 9^th^, 11^th^, 15^th^, 19^th^, 23^th^ and 27^th^ day, respectively. (2) GW9662 group (n = 6): the rats received GW9662 (1 mg/kg/day, i.p, single dose) treatment following the same protocol as adopted in the SB-431542 group. (3) The control group (n = 6) received equal volumes of saline supplemented with the same amount of DMSO (i.p, single dose) following the protocol implemented in the other two groups. The doses and time course of experiments in this study were in the range of those used in other studies involving the same animal species^34^,^35^,^36^. Behavioral analysis, western Blot analysis of c-fos expression in the dorsal horn of the fourth lumbar spinal cord and a-SMA expression in neuromas was performed as in part I and histologic analysis of c-fos expression in the dorsal horn of the fourth lumbar spinal cord was also evaluated (monoclonal rabbit anti-mouse c-fos,1:200, Sigma, USA; secondary monoclonal goat anti-rabbit antibody,1:500, Sigma, USA).

### Behavioral analysis

Autotomy scores of the rats in each group were monitored for 4 weeks and recorded 3 times per week by dual, blinded observers for evidence of neuropathic pain. A modified Wall scale^37^ was used to assign points based on the severity of autotomy. In brief, one point was received for two or more toenails (maximum, one point per limb) and one point was assigned for each half-digit (distal and proximal phalanges) for a possible maximum of 10 points per limb.

### Statistical Analysis

Statistical analyses were carried out using the SPSS.20 statistical software. All values are presented as the means ± standard error of the mean (SEM). Statistical differences were measured with Student’s t-test for comparison between two groups in part I and one-way ANOVA followed by a post hoc comparison test using the LSD method for statistical analysis among groups in part II. Correlation analysis between the expression status of α-SMA and autotomy scores were performed using Pearson’s correlation coefficients and linear regression. P < 0.05 was considered statistically significant.

## Additional Information

**How to cite this article**: Weng, W. *et al.* Significance of alpha smooth muscle actin expression in traumatic painful neuromas: a pilot study in rats. *Sci. Rep.* **6**, 23828; doi: 10.1038/srep23828 (2016).
